# Emergence of Function and Selection from Recursively Programmed Polymerisation Reactions in Mineral Environments†

**DOI:** 10.1002/anie.201902287

**Published:** 2019-07-12

**Authors:** David Doran, Yousef M. Abul‐Haija, Leroy Cronin

**Affiliations:** ^1^ School of Chemistry University of Glasgow Glasgow G12 8QQ UK

**Keywords:** artificial life, chemical recursion, depsipeptides, evolution, polymers

## Abstract

Living systems are characterised by an ability to sustain chemical reaction networks far‐from‐equilibrium. It is likely that life first arose through a process of continual disruption of equilibrium states in recursive reaction networks, driven by periodic environmental changes. Herein, we report the emergence of proto‐enzymatic function from recursive polymerisation reactions using amino acids and glycolic acid. Reactions were kept out of equilibrium by diluting products 9:1 in fresh starting solution at the end of each recursive cycle, and the development of complex high molecular weight species is explored using a new metric, the Mass Index, which allows the complexity of the system to be explored as a function of cycle. This process was carried out on a range of different mineral environments. We explored the hypothesis that disrupting equilibrium via recursive cycling imposes a selection pressure and subsequent boundary conditions on products. After just four reaction cycles, product mixtures from recursive reactions exhibit greater catalytic activity and truncation of product space towards higher‐molecular‐weight species compared to non‐recursive controls.

Biological entities can be viewed as self‐propagating networks of autocatalytic sets in a sustained far‐from‐equilibrium state, in which the stoichiometry of all functionally active components is maintained between generations.^1^ This state is only possible because of the recursive nature of biological replication. Recursive chemical pathways, including biological replication, are defined as those in which the functional, bond‐forming units are regenerated by the pathway and are thus available for further reaction at the end of each reaction cycle.^2^, ^3^ We propose that, for the study of living and potentially life‐like artificial systems in the laboratory, this definition should be refined to include a process of regeneration of chemical systems after disruption of the equilibrium state by dilution and transfer to a fresh environment. Ultimately, biological entities are characterised by the ability to sustain far‐from‐equilibrium states, and regenerate in the presence of what are, relative to the internal components of the system, extremely dilute feedstocks. For the remainder of this Communication, the term “chemical recursion” will be used to refer to a process of continuous dilution of products and their replenishment by fresh feedstocks in a new reaction environment.

Chemical recursion is key not only for maintaining the dynamic, far‐from‐equilibrium states that characterise biology, but also for providing a means of imparting a “chemical history” on generations of cyclical chemical reactions, in which the outcome of one reaction cycle is partly influenced by those that have preceded it. Without such programmed history, there is no possibility for the emergence of evolving chemical systems and therefore life.^4^, ^5^, ^6^ Despite this, much effort has been devoted to non‐recursive syntheses of molecules perceived to be crucial to life's origins, such as RNA.^7^, ^8^, ^9^ The aim of such efforts is to recreate the precise pathways via which the first biomolecules emerged, often under the assumption that heredity and evolution began only after chance accumulation of these molecules.^10^ This may have been confounded by the potentially untestable assumption that a template‐driven, self‐replicating genetic polymer is essential for heredity in the most primitive life or life‐like systems. However, we argue here, as we have done previously,^11^ that recursion is essential from the earliest onset of a living or artificial living system, and that functionality and evolvability can be induced in chemical networks comprised of simpler components than have been considered by many in the origins of life field. Indeed, it is likely that life first emerged from a pool of very simple building blocks. Without strict boundary conditions imposed by recursive selection processes, these building blocks may have reacted in a combinatorial fashion to produce an intractable tar, in which any given product would be far too dilute to impart function. This problem of combinatorial explosion at the earliest onset of life has been discussed previously in the literature.^12^

Experimental frameworks for generating artificial life through recursive selection have been proposed;^1^, ^11^ however, the practical utility of these frameworks is yet to be demonstrated. A major obstacle in achieving such an outcome lies in identifying processes that can steer combinatorial explosions towards a narrower pool of functional products.^13^, ^14^

Confirming whether this has been achieved may be an equally, if not more, daunting task.^15^ In biological systems, a suite of so‐called “omics” technologies enable the surveying of system‐level changes. However, these tools are built around the constrained chemistry of extant biology and have not been designed for studies on artificial life. Hud and co‐workers recently developed a proteomics‐like workflow for sequence mixtures of artificial linear depsipeptides for the first time.^16^ Depsipeptides are mixed oligomers of polypeptides and polyesters, produced from co‐polymerisation of α‐amino acids and α‐hydroxy acids, with a potential for structural and functional diversity that is comparable to that of pure peptides. However, because of the ester–amide exchange that occurs during depsipeptide wet–dry cycling, elongation of these polymers is more thermodynamically favourable at lower temperatures than that of pure peptides.^17^ Nonetheless, structure formation and functional activity in these reactions are yet to be achieved. From a relatively simple starting mixture of four amino acid and α‐hydroxy acid monomers, assuming equal reactivities and a maximum chain length of eight, a potential 65 536 unique depsipeptides can arise. This is excluding branched or cyclic products, which are common for many side‐chain structures, including those used in this study.

We carried out multiple recursive cycles of depsipeptide co‐polymerisation on various mineral substrates in order to 1) assess the robustness of any effects of recursive cycling and 2) provide a means of steering the reaction down different trajectories or “chemical histories”. Three amino acids (l‐leucine, l‐glutamic acid, and l‐lysine) and one α‐hydroxy acid (glycolic acid) were chosen as a model reaction system. A fixed ratio of monomers was reacted on various mineral surfaces in a recursive manner, with approximately 10 % of the product mixture seeded on to fresh mineral and monomer feedstock solutions at the end of each reaction cycle. Nine environments were chosen to steer reaction outcomes: crushed glass, montmorillonite, gypsum, quartz, calcite, chalcopyrite, opal, and kernite, along with a mineral‐free environment. The selected minerals were chosen for their chemical and morphological diversity (see the Supporting Information, Table S3). In previous work, we had examined the ability of minerals and salts to influence the product distribution of similar uncontrolled condensation reactions.^18^ Minerals and other solid substrates have been studied extensively for their ability to concentrate amino acids on their surface, catalyse aqueous peptide polymerisation,^19^, ^20^ and enrich depsipeptides for peptide bonds.^21^ Products were characterised in a functional assay and by mass spectrometry at the end of each reaction cycle (Figure 1). Because of the vast number of potential products, an extensive mass library of over 19 000 linear, cyclic, and branched singly charged species, each corresponding to a unique depsipeptide composition of up to eight‐unit length, was used to screen the mass spectrometry data. Extracted ion chromatograms (EICs) of each potential product composition were obtained for all product mixtures. The Mass Index, which normalises the number of observed product compositions to their mass distribution, was used as a simple, objective criterion for assessing differences between product mixtures (Figure 2). The library used for screening comprised 6633 depsipeptide compositions with various common adducts, giving a total of 19 899 masses. This compositional screening method covers a significant portion of feasible product space. However, it is pertinent to note that while use of branching monomers widens the vast gamut of potential products, it precludes sequencing of distinct branched sequences with identical compositions.

**Figure 1:**
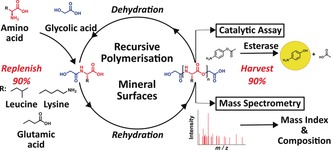
Glycolic acid mediated peptide bond formation and depsipeptide elongation by ester–amide exchange. This is repeated in an iterative fashion with fresh addition of starting materials on various solid surface matrices. Compositional and functional analyses were carried out at the end of each reaction cycle by mass spectrometry and functional assays, respectively.

**Figure 2:**
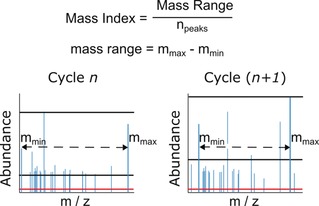
Mass spectrometry data processing workflow. Raw data is filtered to remove any MS^1^ hits not in the depsipeptide product library. An absolute noise threshold (red line) is applied to all remaining peaks. To calculate the Mass Index, a further filtering step is applied, with all species with a total abundance of <4.55 % of the most abundant species discarded. The mass range of remaining peaks is then normalised to the number of peaks within this intensity range (black lines).

To calculate the Mass Index, raw mass spectrometry data was filtered using a depsipeptide mass library and an absolute noise threshold. After this, a second threshold of relative intensity was applied, retaining only species that exceeded 4.55 % of the most intense/abundant species in the mass library. The mass range of species within this intensity range was then divided by the number of remaining species. Thus, an increase in the Mass Index reflects a funnelling of product space from a large pool of low‐molecular‐weight species to a smaller product pool with greater relative abundance of higher‐mass products. We observed a clear increase in the Mass Index over recursive cycles in the mineral‐free environment while the non‐recursive controls showed a much smaller increase (Figure 3 a). Differences between the various mineral environments were also observed using this metric. However, broadly similar trends were observed, with seven of nine environments showing a clear increase in the Mass Index after four recursive cycles (Figure S3), demonstrating the robust effects of chemical recursion in this system.

**Figure 3:**
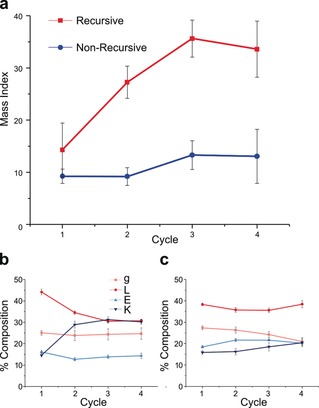
Change in depsipeptide product length distribution and as observed via the Mass Index (a) and relative monomer composition of products (b and c for recursive and non‐recursive samples in a mineral‐free reaction environment, respectively). “g”=glycolic acid; “L”=leucine; “E”=glutamic acid; “K”=lysine. Data represent means of 9 replicates ±1 standard deviation.

The relative monomer composition of the products was also observed from the mass spectrometry data, and a trend towards increasing lysine content over four cycles correlated with a depletion of leucine in products (Figures 3 b and S4). This trend was not observed in the non‐recursive control samples (Figures 3 c and S4). No evidence of glycolic acid depletion was found in the recursive reactions, perhaps due to continuous feeding of glycolic acid in the replenishing monomer stock.^22^ However, a decrease in glycolic acid content was observed for most of the cyclic, non‐recursive control reactions, consistent with the peptide enrichment found in cyclical depsipeptide elongation in literature reports.^3^, ^16^

Having observed the effect of recursion on the composition of depsipeptide products, the next step was to test for the emergence of function. A hydrolytic assay was used to determine the esterase activity of products. Ester bond hydrolysis is an important step in the ester–amide exchange reaction that enables depsipeptide elongation,^3^ and a vital process in biology. *p*‐Nitrophenyl acetate (*p*NPA), which breaks down into yellow *p*‐nitrophenol (*p*NP) and acetate, was used as an esterase substrate for the hydrolytic assay.^23^ The activity of product mixtures in this assay was compared and normalised to a standard curve of α‐chymotrypsin (Figures 4 and S6), and activity was measured in esterase units per mL of product solution. Recursive product mixtures from the mineral‐free, montmorillonite, quartz, calcite, and chalcopyrite environments exhibited a sharp increase in activity after four cycles (Figure 4). This increase was reproducible, but not stepwise, occurring sharply between cycles 3 and 4. No comparable increase was observed in the non‐recursive control reactions (Figures 4 and S7).

**Figure 4:**
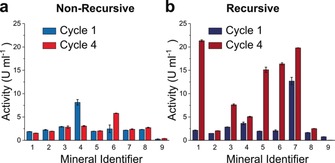
Effect of solid surface matrices on esterase activity. Esterase activity (in enzyme units, U, per mL) for a) non‐recursive and b) recursive products after 1 and 4 reaction cycles on the following mineral environments: 1) no mineral; 2) glass; 3) montmorillonite; 4) gypsum; 5) quartz; 6) calcite; 7) chalcopyrite; 8) opal; 9) kernite. Data represent means of 2 replicates ±standard deviation.

The increase in esterase activity with chemical recursion suggests some form of selective advantage of functionally active products in a recursive system. Interestingly, this also correlates with the increase in lysine and depletion of leucine content of products (Figures 3 b, S5, and S6). Lysine is one of the most functionally active amino acids in modern biochemistry—as well as taking part in acid–base catalysis, lysine is readily modified because of the additional amine sidechain.^24^ It is unclear at this stage whether lysine enrichment of depsipeptides plays a role in our observed increase in esterase activity.

Structural characterisation in solution was carried out to assess any impact of this change in composition on secondary structure formation in products. Circular dichroism profiles of mixtures differed markedly from starting material controls, with a characteristic β‐sheet signal at 210 nm, indicating potential secondary structure formation consistent with lysine‐rich sequences.^25^ However, no evidence was found of this signal increasing after lysine enrichment via recursive cycling (Figure S10). Similarly, two FTIR absorption bands were identified at 1600 and 1650 cm^−1^ in products, possibly corresponding to the amide I and amide II structural bands, respectively.^26^ Starting material controls exhibited only one band in this region, at approximately 1625 cm^−1^, most likely corresponding to free carboxyl groups of α‐amino and α‐hydroxy acids. Further investigation would be required to determine whether absorption bands in products represent secondary structure formation (Figure S11).

In this work, the emergence of selection and functionality from unconstrained, recursive depsipeptide polymerisation has been demonstrated for the first time. Further work is ongoing to determine the precise mechanism of this phenomenon. Recursive cycling was used to induce a selection pressure on the system. The ability of mineral surfaces to selectively stabilise small molecules is well documented, as is the heat lability of many depsipeptides.^3^ Thus, only those products capable of persisting under continuous heating at 90 °C in their reaction environment would have been selected for measurement and participation in further cycles. Nevertheless, the successful truncation of product space towards higher‐mass products through recursive cycling persisted in all but two mineral environments tested for this system, with a majority of environments also facilitating increased catalytic activity of products after just four reaction cycles. This demonstrates the potential importance of chemical recursion in truncating combinatorial explosion, which is essential for complex, functional species to arise in sufficient abundance to aid the transition from non‐living to living systems. Future work will also demonstrate the applicability of the Mass Index in the screening of sequence libraries, and also screening sequence space for sub‐populations of products that are responsible for esterase activity and structure formation. This will require the development of new techniques for the sequencing of multiply branched depspeptides. The ultimate goal of such work is to elucidate principles governing the emergence of functionality and selection from unconstrained “messy” chemistry.

## Conflict of interest

The authors declare no conflict of interest.

## Supporting information

As a service to our authors and readers, this journal provides supporting information supplied by the authors. Such materials are peer reviewed and may be re‐organized for online delivery, but are not copy‐edited or typeset. Technical support issues arising from supporting information (other than missing files) should be addressed to the authors.

Supplementary
