# Supplementary material for: Emergence of Function and Selection from Recursively Programmed Polymerisation Reactions in Mineral Environments
Source: Angew Chem Int Ed Engl. 2019 Jul 12;58(33):11253–6. doi: 10.1002/anie.201902287 (PMC6772075; doi:10.1002/anie.201902287)
Supplement: Supplementary file 1 — Supplementary [file ANIE-58-11253-s001.pdf]

Angewandte Chemie
Eine Zeitschrift der Gesellschaft Deutscher Chemiker

Supporting Information

Emergence of Function and Selection from Recursively Programmed Polymerisation Reactions in Mineral Environments**

*David Doran, Yousef M. Abul-Haija, and Leroy Cronin**

anie_201902287_sm_miscellaneous_information.pdf

Author Contributions

L.C. conceived the initial idea, experimental design including the metric, and coordinated the team with Y.M.A.-H. D.D. carried out all reactions, mass spectrometry analysis and structural and functional assays, and wrote Python 3.6 scripts for adduct screening and calculating monomer composition. Y.M.A.-H. was instrumental in guiding the work, providing expertise in peptide chemistry and, together with D.D., developed project ideas. D.D., Y.M.A.-H., and L.C. wrote the manuscript.

1. **Depsipeptide Polymerisation……..……… 2**
2. **Mass Spectrometry…………………………………………………………..2**
   2.1 Mass Spectrometry Data Acquisition………………………………………..2
   2.2 Depsipeptide Library Screening and Mass Index………………………….3
   2.3 Product Composition…………………………………………………….....6
3. **Esterase Activity Assay………………………………………………………. 11**
4. **Structural Characterisation………………………………………………..... 13**
   4.1 Circular Dichroism…………………………………………………. 13
   4.2 Fourier Transform Infrared Spectroscopy……………………….. 14
5. **Minerals……………………………………………………………………….. 16**
6. **Wet-Dry Cycling Reactions…………………………………………………….. 16**

1. Depsipeptide Polymerisation

10 ml solutions of 30 mM L-leucine (Sigma, CAS: 61-90-5), 30mM L-glutamic acid (Sigma, CAS: 56-86-0), 30 mM L-lysine (Sigma, CAS: 56-87-1) and 100 mM glycolic acid (Sigma, CAS: 79-14-1) in HPLC-grade H_2O were adjusted to pH 2.5 using H_3PO_4 and heated at 90 $^{\circ}$C for 15 hours in open cap glass vials on a 75 vial insert heating slab (Figure S12). For recursive mineral reactions, 150 mg of solid mineral matrix was added to the vials at the beginning of the first reaction cycle. After 15 hours of heating, the reaction solutions had completely evaporated, and products were re-suspended in 10 ml HPLC-grade H_2O. Re-suspended product was vortexed, and 1 ml of product solution was transferred to 9 ml fresh reagent solution and 135 mg fresh solid matrix in a fresh vial. Solutions were re-adjusted to pH 2.5, and the process was repeated for each subsequent cycle. For non-recursive control reactions, the first cycle was carried out as in the recursive reactions; however, products were not transferred to fresh feedstocks and no further solid matrix material was added to the mixture. Montmorillonite, gypsum, quartz, calcite, chalcopyrite, opal and kernite solid matrix materials were sourced from Richard Tayler minerals. Solids were crushed and passed through a sieve with a 3 µm cut-off prior to addition to the reaction. Crushed glass was generated using the same process from the same glass vials that were used for all reactions.

2. Mass Spectrometry

2.1 Mass Spectrometry Data Acquisition

Product mixtures were diluted 1:100 in HPLC-MS grade H_2O, filtered through a nylon membrane with 0.2 µm pore size into glass vials (#2-SVW8-CPK, ThermoScientific) and loaded on to an autosampler (#WPS-3000TRS, ThermoScientific) hooked up to a quaternary pump (#LPG-3400RS, ThermoScientific). Samples were injected in 10 µl aliquots into a Bruker Maxis Impact II in a 1 ml min^{-1} flow of HPLC-MS grade H_2O + 0.1% formic acid (Sigma, CAS: 64-18-6). Measurements were taken in positive ion mode, with the instrument calibrated to a range of 50-1200 *m/z* using sodium formate calibrant solution. Voltage of the capillary tip was set to 4800 V, end plate offset at -500 V, funnel 1 RF and funnel 2 RF at 400 Vpp, hexapole RF at 100 Vpp, ion energy at 5.0 eV, collision energy at 5 eV, collision cell RF at 200 Vpp, transfer time at 100.0 µs and pre-pulse storage time at 1.0 µs.

2.2 Depsipeptide Library Screening and Mass Index

The complete mass list of 6363 depsipeptide products was compiled in Python 3.6. All possible compositions arising from leucine (L), glutamic acid (E), lysine (K) and glycolic acid (g) were calculated from a dictionary of monomer masses read from a .json file. Branched and cyclic products were screened for by removing one water mass (18.01056 amu) for every two proposed branching points. Cationic adducts of each branched, cyclic and linear product were accounted for by adding the following masses: 1.007276 (H^+), 22.989 (Na^+), 38.963 (K^+), 18.034 (NH_4^+). The mass list was compiled as a Python dictionary, with keys corresponding to a string of monomer units plus dehydrations and adducts. Dehydrations were added in the string format "- <n> H2O" and adducts were added in the string format "+ <i>", where n and i correspond to number of dehydrations and cationic adduct, respectively. For example, "LEK – 1 H_2O + Na" corresponds to a trimer of one L, one E and one K monomer with one extra dehydration (a potential additional cycling or branching point) plus a sodium adduct in +1 charge state.

```
{
  "E": 129.043,
  "K": 146.10557,
  "L": 131.09467,
  "g": 76.0160
       }
```

Figure S1: Monomer Unit Mass Dictionary. Each potential monomer unit plus masses were stored as a dictionary, with keys corresponding to standard amino acid one letter codes (upper case) or lower-case letter "g" corresponding to the glycolic acid monomer unit. Standard, upper case one letter codes were used for amino acids: "E" = glutamic acid; "K" = lysine; "L" = leucine.

```
n_dhr = 8
monomers = ["L", "E", "K", "g"]
# define paths to input and output JSON file
input_file_path = "C:\\Users\croningp\PycharmProjects\Mass_Spec_Mass_Lists\Amino Acid Mass Dictionary.json"
output_file_path_full = "C:\\Users\croningp\Documents\MS Analysis\Mass Lists\DEPSI-Peptide\{0}{1}{2}_13012018.json".format(monomers[0], monomers[1], monomers[2])
dhr_output_file_path_full = "C:\\Users\croningp\Documents\MS Analysis\Mass Lists\DEPSI-Peptide\{0}{1}{2}{3}dhr13012018.json".format(monomers[0], monomers[1], monomers[2], n_dhr)

# define maximum oligomer length
max_length = 8
water = 18.010565
H = 1.007276

adduct_dict = {"Na": 22.989218, "K": 38.963158, "NH4": 18.033823}
```

Figure S2: Mass List Compiler. Maximum number of dehydrations per oligomer is set by variable n_dhr, monomers used for compilation are added to monomers list, and read from monomer mass dictionary (Figure S1). Maximum length of oligomers is set by max_length variable. Masses of water and H^+ adduct are defined by water and H variables. Additional adducts are stored as a dictionary adduct_dict, with corresponding masses.

Mass lists in .csv format were read by a script written in R 2.7 and run in R-studio. Extracted ion chromatograms (EICs) for each mass were extracted from MS-1 data stored in 32-bit mzml files, which were generated from raw data using Proteowizard MS Convert. Masses were extracted with an absolute error threshold of + / - 0.01. Total intensities for each EIC were stored in csv files. The first column of each csv file contained a list of depsipeptide composition strings, each subsequent column contained corresponding intensities for depsipeptide products for each sample. A small example data set is presented in Table S1. A noise threshold of 4.55% of maximum intensity was applied to this data. Noise filtered data was used for the Mass Index measurement.

Table S1: Example Data Set for Mass Index Measurement**.** The top row of each column contains headers, the first of which denotes the column containing m/z values; each subsequent column header denotes the name of a sample measurement file. The first column (left) contains a list of *m/z* values, each corresponding to a species in the mass library. Subsequent columns contain intensities obtained in the mass spectrometry measurement of each sample corresponding to that *m/z* value.

**m/z**	**No Mineral_Cycle1_A**	**No Mineral_Cycle1_B**	**No Mineral_Cycle1_C**
130.049	266141.711	271363.245	249274.3635
132.101	864710.269	863549.7875	854975.3445
135.029	0	0	0
136.076	0	0	0
139.003	0	0	0
147.112	328543.4595	314114.8865	285210.6015
148.060	166482.322	152707.5075	140961.1295
151.087	0	0	0
152.034	62206.188	56934.0725	57648.0905

The Mass Index was used as a metric for assessing the effect of recursion on the reaction carried out in nine mineral environments (Figure S3).

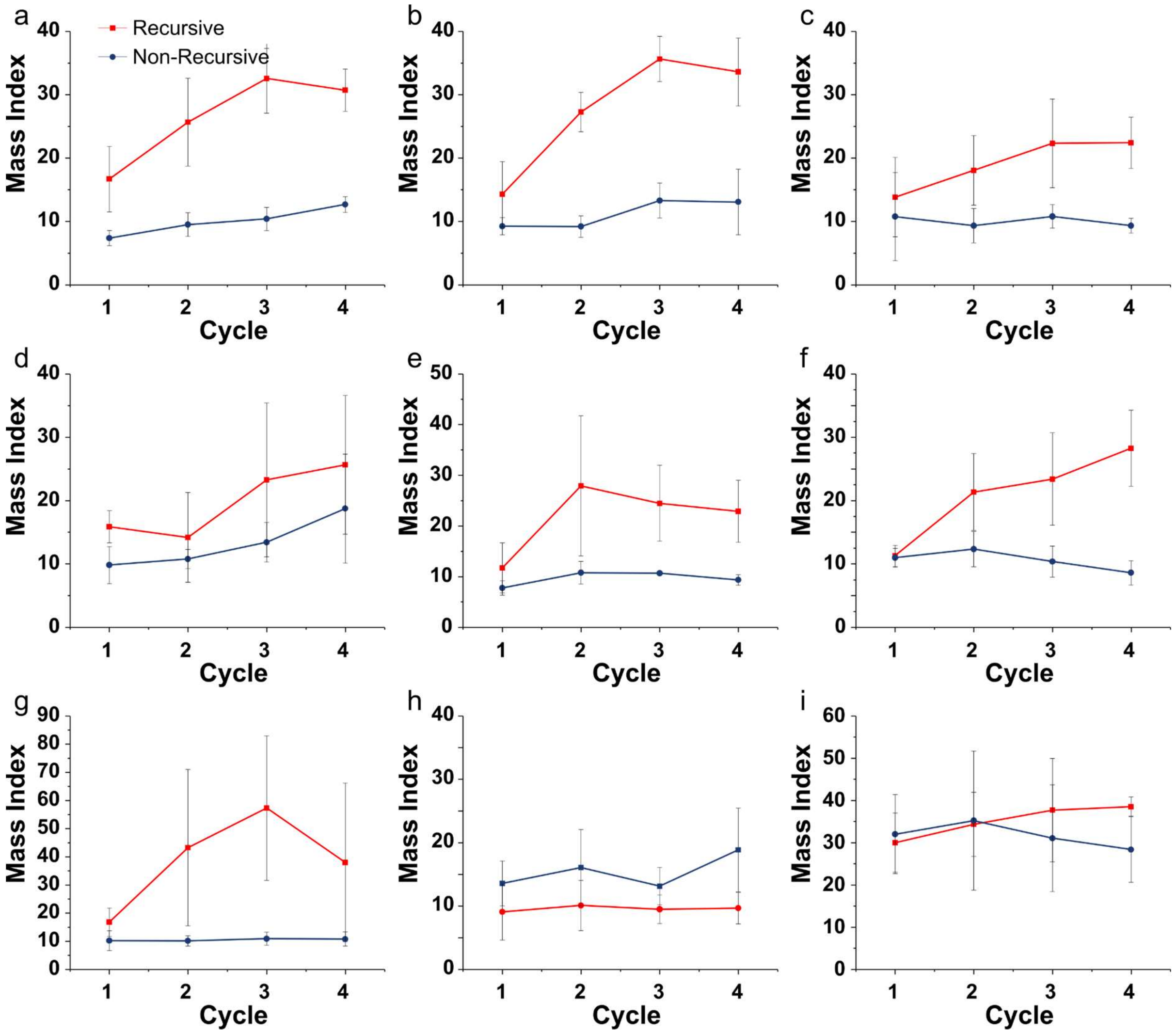

Figure S3. Mass Index by cycle for recursive and non-recursive depsipeptide product mixtures reacted on various solid surface matrices: A) no mineral; B) crushed glass; C) montmorillonite; D) gypsum; E) quartz; F) calcite; G) chalcopyrite; H) opal; I) kernite. Data represent mean of 9 replicates + / - 2 S.D.

2.3 Product Composition

Monomer composition measurements (Figure 3, Supplementary Fig. 4) were obtained from csv files containing depsipeptide composition strings and their corresponding intensities. The following steps were carried to extract monomer composition:

1. Iteration through strings corresponding to depsipeptide compositions, removing extra adducts but keeping dehydrations.
2. For each composition product, addition of the intensities for each adduct.
3. For each composition string, calculation of the relative ratio of each monomer string ("L", "E", "K", "g") as a decimal fraction, excluding extra string characters for dehydration and whitespace.
4. Multiplication of each of these fractions by the total intensity measured for the composition product.
5. Addition of total monomer intensities over the entire library of 6363 compositions.
6. For each total monomer intensity, division by the total product intensity and multiplication by 100 to obtain % monomer intensity.

An example of csv data input into the monomer composition script is presented in Table S2. Monomer composition values for mineral environments not shown in the main paper (Figure 3) are given below, in Supplementary Fig. 4.

Table S2: Combined Adduct Intensity Data for Monomer Composition Extraction**.** Top table shows depsipeptide strings corresponding to a leucine (L) trimer with one extra dehydration plus adducts (K, NH_4 and Na) with their corresponding intensity values for three samples. Lower table (highlighted in yellow) shows the combined intensity of all adduct species. These combined intensity values were used to calculate percentage intensity contribution of monomer species (66.67 % and 33.33 % for L and g, respectively). L intensities: 128390.323 (sample 1), 115573.041 (sample 2), 104842.845 (sample 3). g intensities: 64195.161 (sample 1), 57786.520 (sample 2), 52421.422 (sample 3).

Product Composition	Sample 1	Sample 2	Sample 3
LLg -1 H_2O	119798.404	94274.3355	85355.094
LLg -1 H_2O + K	74732.388	80836.3325	73497.701
LLg -1 H_2O + NH4	0	0	0
LLg -1 H_2O + Na	0	0	0

Product	Sample 1	Sample 2	Sample 3
LLg-1 H_2O	194530.792	175110.668	158852.795

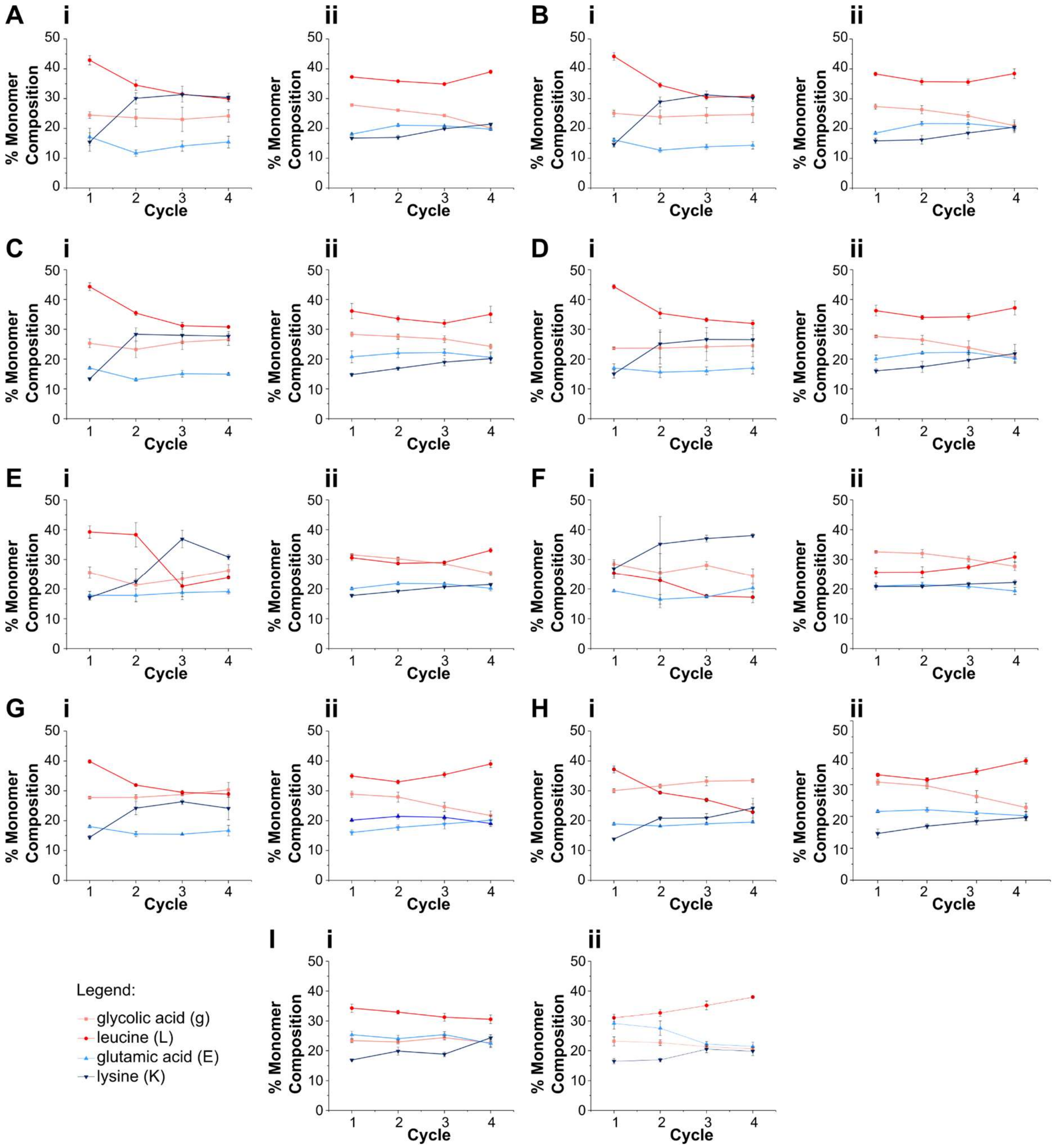

Figure S4: Monomer Composition of Depsipeptide Products over Multiple Recursive Cycles. Intensity contribution to products as measured by mass spectrometry are shown for depsipeptide reactions carried out on: **A)** no mineral; **B)** crushed glass**; C)** montmorillonite; **D)** gypsum; **E)** quartz; **F)** calcite; **G)** chalcopyrite; **H)** opal; and **I)** kernite for (i) recursive and (ii) non-recursive products. Data points represent the mean of 9 replicates + / - 1 S.D. g = glycolic acid; L = leucine; E = glutamic acid; K = lysine.

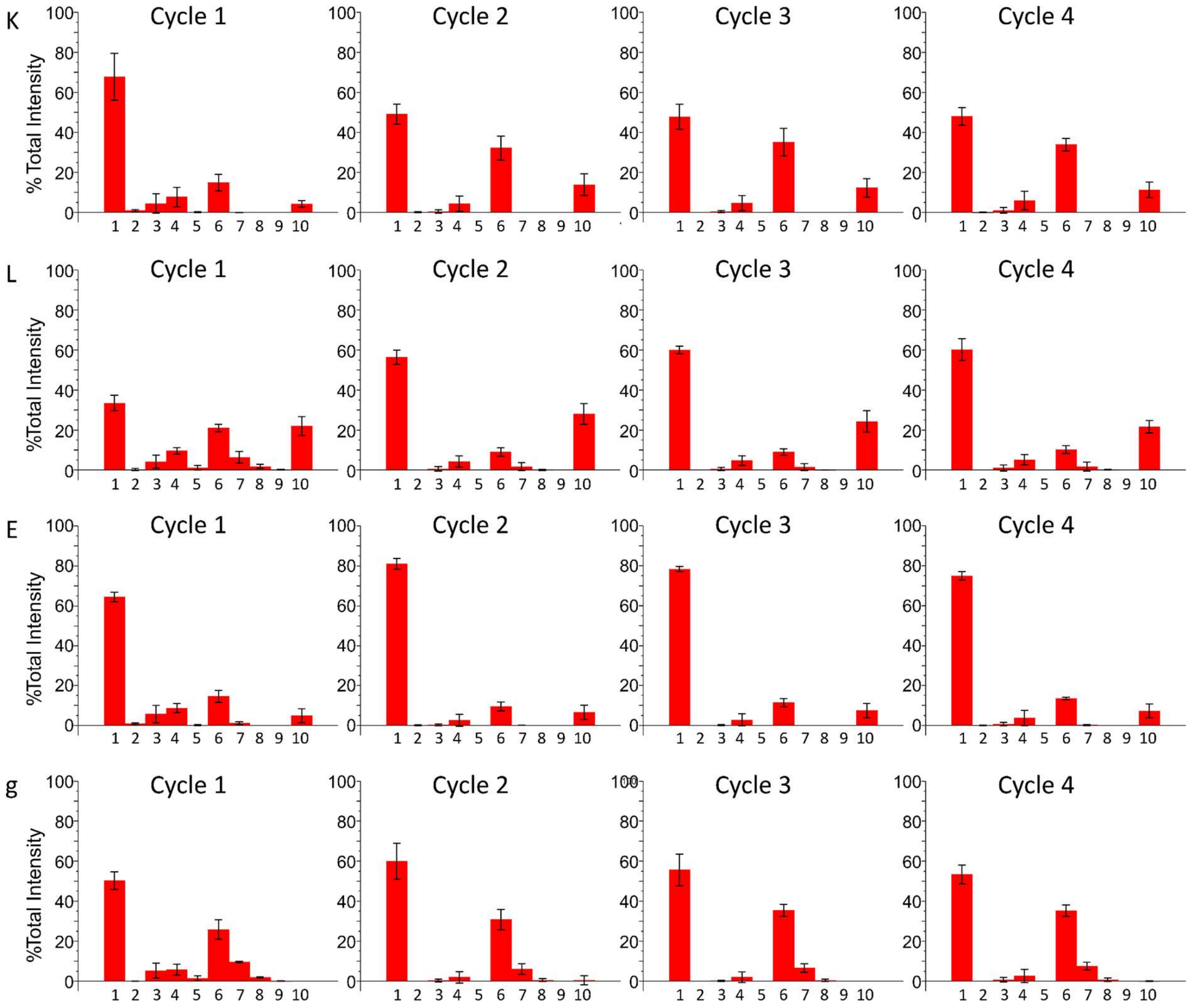

Figure S5: Monomer Distribution in Products for Mineral-free Recursive Reactions. Intensity contribution of products for lysine (K), leucine (L), glutamic acid (E) and glycolic acid (g) at cycles 1, 2, 3 and 4 for the following monomer compositions: 0-10% (1), 10-20% (2), 20-30% (3), 30-40% (4), 40-50% (5), 50-60% (6), 60-70% (7), 70-80% (8), 80-90% (9), 90-100% (10). Data represent mean of nine measurements + / - 1 S.D.

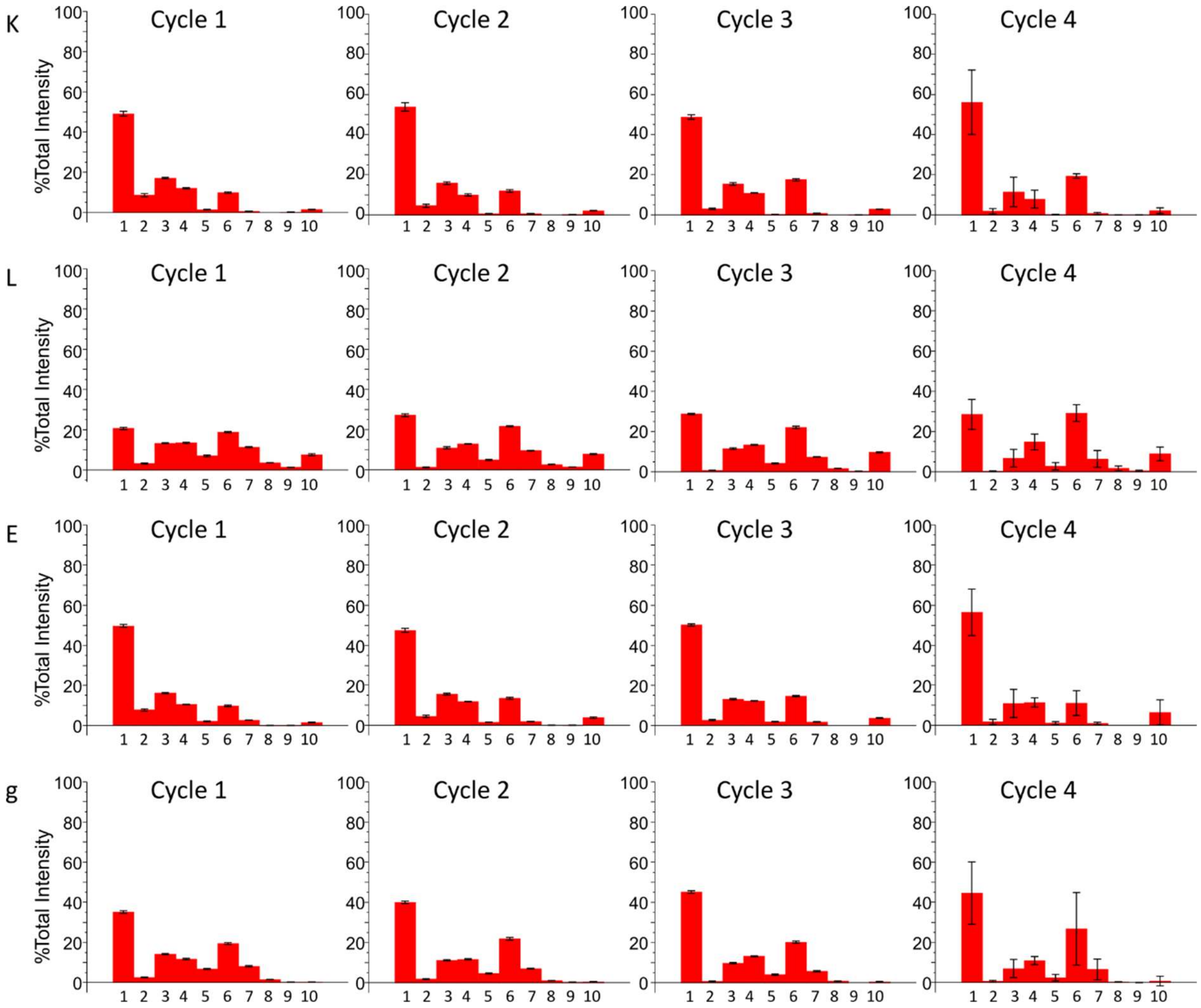

Figure S6: Monomer Distribution in Products for Mineral-free Non-Recursive Reactions. Intensity contribution of products for lysine (K), leucine (L), glutamic acid (E) and glycolic acid (g) at cycles 1, 2, 3 and 4 for the following monomer compositions: 0-10% (1), 10-20% (2), 20-30% (3), 30-40% (4), 40-50% (5), 50-60% (6), 60-70% (7), 70-80% (8), 80-90% (9), 90-100% (10). Data represent mean of nine measurements + / - 1 S.D.

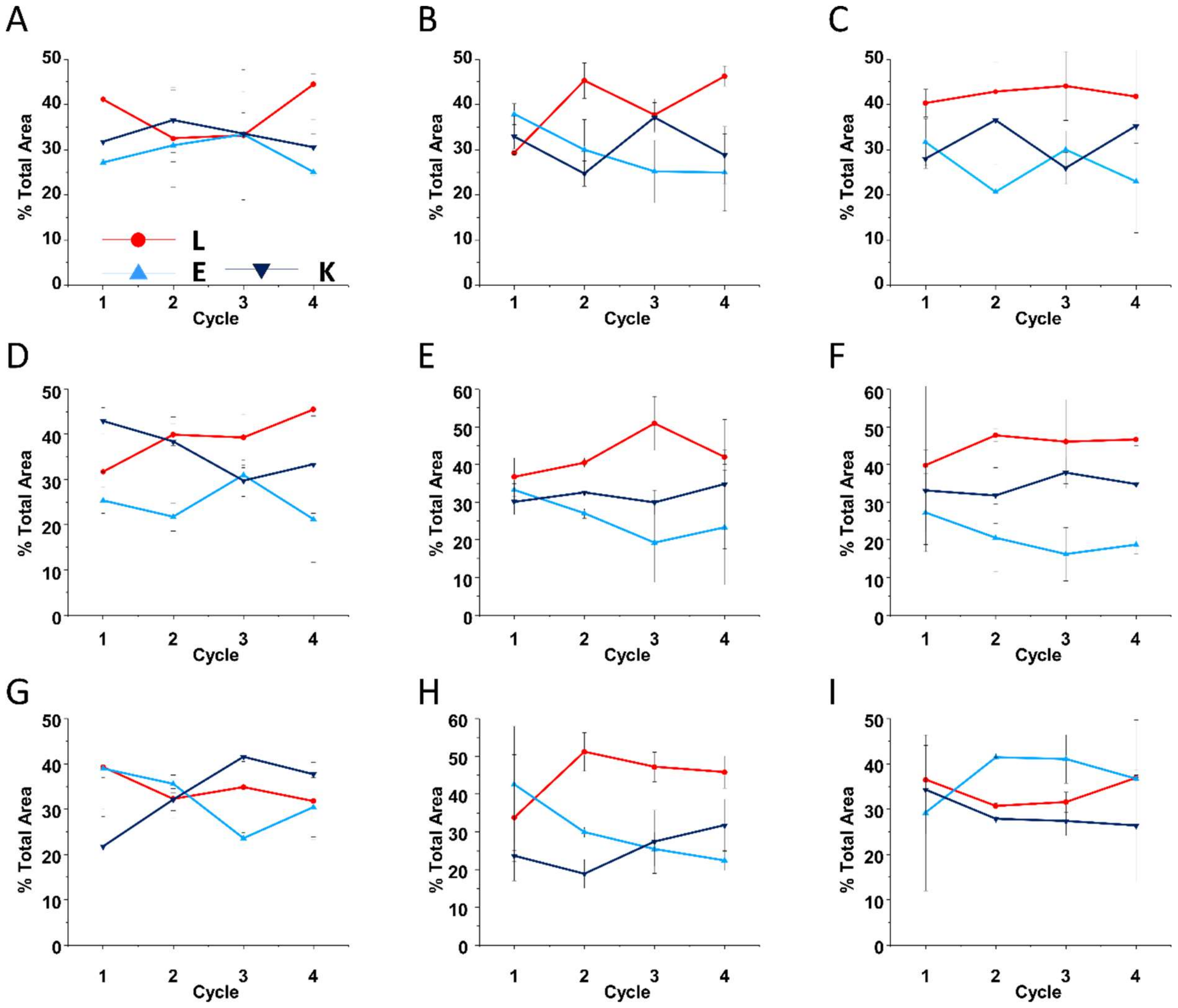

Figure S7: Relative Free Amino Acid Concentration over Multiple Recursive Cycles. Relative concentration of free Leucine (L), glutamic acid (E) and lysine (K) are shown for depsipeptide reactions carried out on: **A)** no mineral, **B)** crushed glass, **C)** montmorillonite, **D)** gypsum, **E)** quartz, **F)** calcite, **G)** chalcopyrite, **H)** opal, **I)** kernite.

3. Esterase Activity Assay

p-nitrophenyl acetate (pNPA) (CAS: 830-03-5) and α-chymotrypsin standards containing 40 enzyme units (U) per mg (CAS: 9004-07-3) were purchased from Sigma. Standard solutions were made up by serial dilution in cold (4 °C) 10 mM HCl. Standards were run at the following concentrations (U per ml): 40, 20, 10, 5, 2.5, 1.25. Prior to running on the assay, 1 ml aliquots of product solutions were adjusted to pH 7 *via* addition of aqueous NaOH and centrifuged at 4000 rpm for at least 5 minutes. To each well on a 96-well plate, 50 µL of product supernatant or chymotrypsin standard and 150 µL of buffered 130 µM *p*NPA solution was added. All standards and samples were run in duplicate. *p*NPA hydrolysis was monitored by measuring absorbance at 405 nm. Measurements were taken every 2 minutes for 2 hours using a Tecan Infinite® 200 Pro plate reader.

Esterase activity of product mixtures was normalised to an α-chymotrypsin standard curve (Supplementary Fig. 8a). Rate of *p*NPA hydrolysis was measured by rate of increase in absorbance at 405 nm (Supplementary Fig. 8b). To calculate the esterase activity of products, rate of increase in absorbance at 405 nm was substituted into the equation of the standard curve fit in Supplementary Fig. 8A.

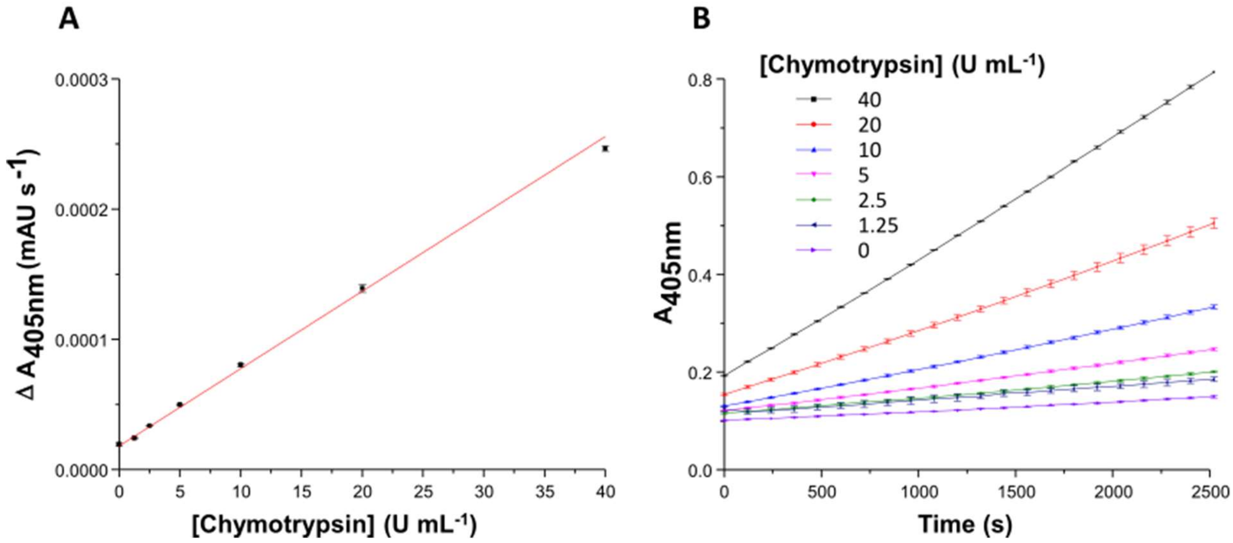

Figure S8: Chymotrypsin Standards for *p*NPA Hydrolytic Assay. A) Rate of *p*NPA hydrolysis (measured by increase in absorbance at 405 nm) plotted for α-chymotrypsin standards at 40, 20, 10, 5, 2.5, 1.25 and 0 enzyme units (U) per ml. Equation of curve fit: $y = mx + c$, where $y = \Delta A_{405\ nm}$, $m = 5.9793 \times 10^{-6}$, $x =$ [chymotrypsin], $c = 1.82694 \times 10^{-5}$. B) Raw data from *p*NPA hydrolytic assay for α-chymotrypsin standards showing absorbance at 405 nm over time.

The esterase activities of all recursive and non-recursive product mixtures were ran for cycles 1-4. Recursive product mixtures showed a trend towards increased activity from cycles 1 to 4 (Supplementary Fig. 9). Esterase activity of product mixtures exceeded that of the controls, and also unreacting starting material (main text Figure 4), demonstrating that *p*NPA hydrolysis was not due to hydrolysis by H_3PO_4 or monomers.

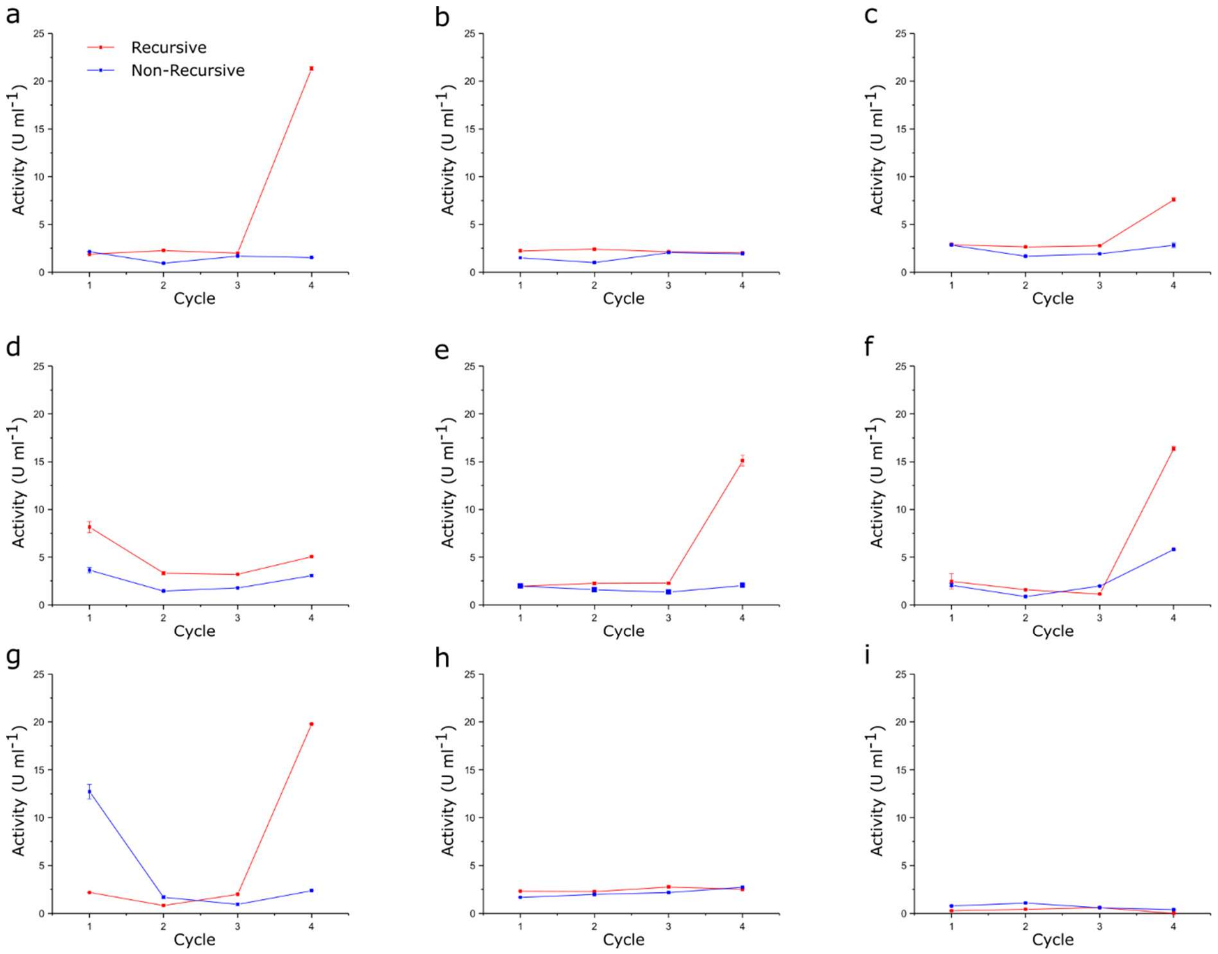

Figure S9: Esterase Activity of Depsipeptide Products Measured by *p*NPA Hydrolytic Assay. Activity was measured in enzyme units (U) per ml for product mixtures after 1-4 cycles carried out on: A) no mineral; B) crushed glass; C) montmorillonite; D) gypsum; E) quartz; F) calcite; G) chalcopyrite; H) opal; I) kernite for both recursive (red) and non-recursive (blue) product mixtures. Data show mean of 2 replicates + / - 2 S.D.

4. Structural Characterisation

4.1 Circular Dichroism

Circular dichroism (CD) was used to screen for secondary structures in recursive product mixtures. CD spectra of product mixtures were taken after cycles 1, 3 and 5, and compared to unreacted starting material.

Product mixtures were diluted 1:1 in pH 8.0 sodium phosphate buffer (93 mM Na_2HPO_4, 7 mM NaH_2PO_4) in HPLC-grade H_2O, vortexed and sonicated at 45 $^{\circ}C$ for approximately 15 minutes, before chilling at 4 $^{\circ}C$ overnight. Immediately prior to CD measurements, buffered product mixtures were diluted 1:20 in fresh pH 8 buffer solution. CD measurements were taken at room temperature using a Jasco J-810 spectropolarimeter in a 0.2 cm path with a data pitch of 0.1 nm, in continuous scanning mode at a scan speed of 100 nm min^{-1}, 2 second response time, accumulation of 1 and bandwidth of 1 nm. Spectra were recorded from 190-400 nm.

Data in Supplementary Fig. 10 show some form of secondary structure - possibly β-sheets, which are common for lysine-rich sequences and also hydrophobic peptides.

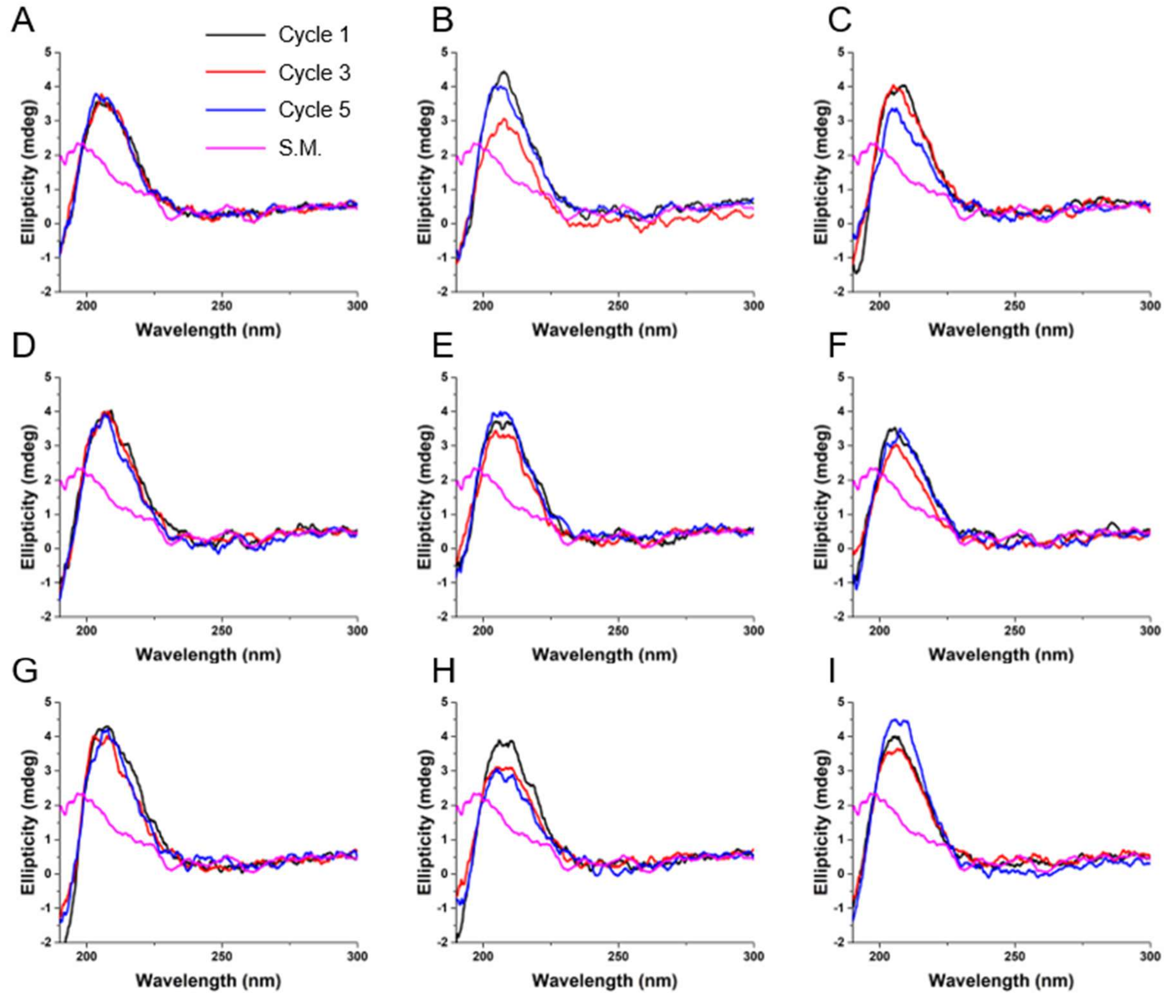

Figure S10: Circular Dichroism of Recursive L, E, K and g Depsipeptide Products. CD measurements were taken of products from cycles 1, 3 and 5 and unreacted starting material (S.M.) mixture at equivalent concentration from A) no mineral; B) crushed glass; C) montmorillonite; D) gypsum; E) calcite; F) chalcopyrite; G) opal; H) kernite. β-sheet profile is found in all product mixtures, but not unreacted starting material.

4.2 Fourier Transform Infrared Spectroscopy

FTIR measurements (Supplementary Fig. 11) were taken to confirm the presence of secondary structures measured by CD.

1 ml of product mixture was diluted 1:1 in HPLC-grade H_2O, frozen at -80 °C and lyophilised using a Christ™ Alpha 1-2 LDplus freeze-dryer. Lyophilised product material was then re-suspended in 1 ml deuterated pH 8 sodium phosphate buffer (93 mM Na_2HPO_4, 7 mM NaH_2PO_4). Deuterated water (CAS: 7789-20-0) was purchased from Sigma. Fourier transform infrared spectroscopy (FTIR) measurements were taken using an IRAffinity-1S FTIR

spectrophotometer (Shimadzu). 64 scans were taken for each measurement, with resolution set at 16 cm^{-1} for a spectral range of 2000 cm^{-1} to 600 cm^{-1}.

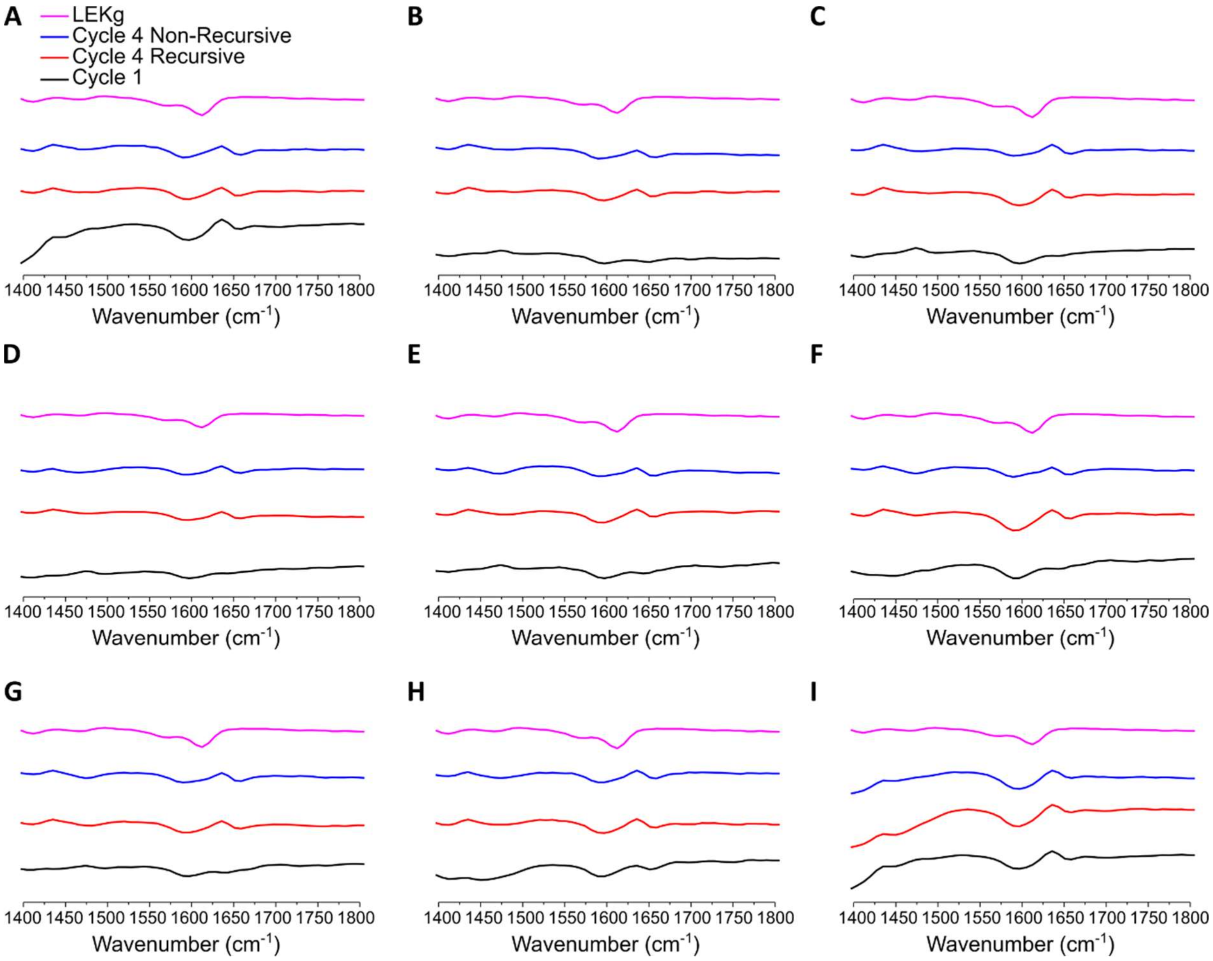

Figure S11: Fourier Transform Infra-Red (FTIR) Spectroscopy of Depsipeptide Mixtures. FTIR absorption spectra of product mixtures are shown after 1 cycle (black) and 4 recursive (red) and non-recursive (magenta) cycles, with starting material control (magenta) of L-Leucine (L), L-glutamic acid E, L-Lysine (K) and glycolic acid (g). A = no mineral; B = crushed glass; C = montmorillonite; D = gypsum; E = quartz; F = calcite; G = chalcopyrite; H = opal; I = kernite. Data represent mean of 2 measurements + / - 2 S.D.

5. Minerals

Minerals were sourced from Richard Tayler minerals. Composition, structure and water solubility are given below in Table S3.

Table S3: Chemical and Physical Properties of Minerals used for Solid Surface Matrices.

Mineral	Composition	Structure	Water Soluble?
Montmorillonite	$(Na,Ca)_{0.33}(Al,Mg)_2(Si_4O_{10})(OH)_2{\cdot}nH_2O$	Crystalline - monoclinic prismatic	No
Gypsum	$CaSO_4{\cdot}2H_2O$	Crystalline - monoclinic prismatic	2-2.5 g / L
Calcite	$CaCO_3$	Crystalline - hexagonal scalenohedral	Highly
Chalcopyrite	$CuFeS_2$	Crystalline - tetragonal scalenohedral	No
Quartz	SiO_4	Crystalline - trigonal or hexagonal	No
Opal	$SiO_2{\cdot}nH_2O$	Amorphous	No
Crushed glass	SiO_2	Amorphous	No
Kernite	$Na_2B_4O_6(OH)_2.3H_2O$	Crystalline - monoclinic prismatic	Yes

6. Wet-Dry Cycling Reactions

Wet-dry cycling reactions were carried out in 15 ml glass vials placed on to 75-well heating slabs (Supplementary Fig. 12). The temperature of the heating slabs was set to and maintained at 90 °C *via* a custom PID controller designed and built in-house, the ThermoShield. Fine power control of resistive loads such as the silicone heating mat employed in this project is achieved via phase angle control utilizing a TRIAC. An EPCOS B57560G104F NTC Thermistor submerged in a vial filled with mineral oil (CAS: 8042-47-5, Sigma) was used to monitor the current temperature. A custom PCB featuring circuitry for reading the NTC, sensing the AC zero crossing, and firing the TRIAC was designed to fit on top of an Arduino Mega 2560 microcontroller board. A simple firmware utilizing the Arduino PID library (http://playground.arduino.cc/Code/PIDLibrary) provides communication and control. Since no complicated temperature programs were required, the ThermoShield was controlled through the terminal program Termite (https://www.compuphase.com/software_termite.htm). Temperature was monitored both through Termite and through a thermometer placed in the same vial.

Gerber files, Bill of Materials, Arduino sketch and documentation can be found on github: http://datalore.chem.gla.ac.uk/Origins/ThermoShield.git

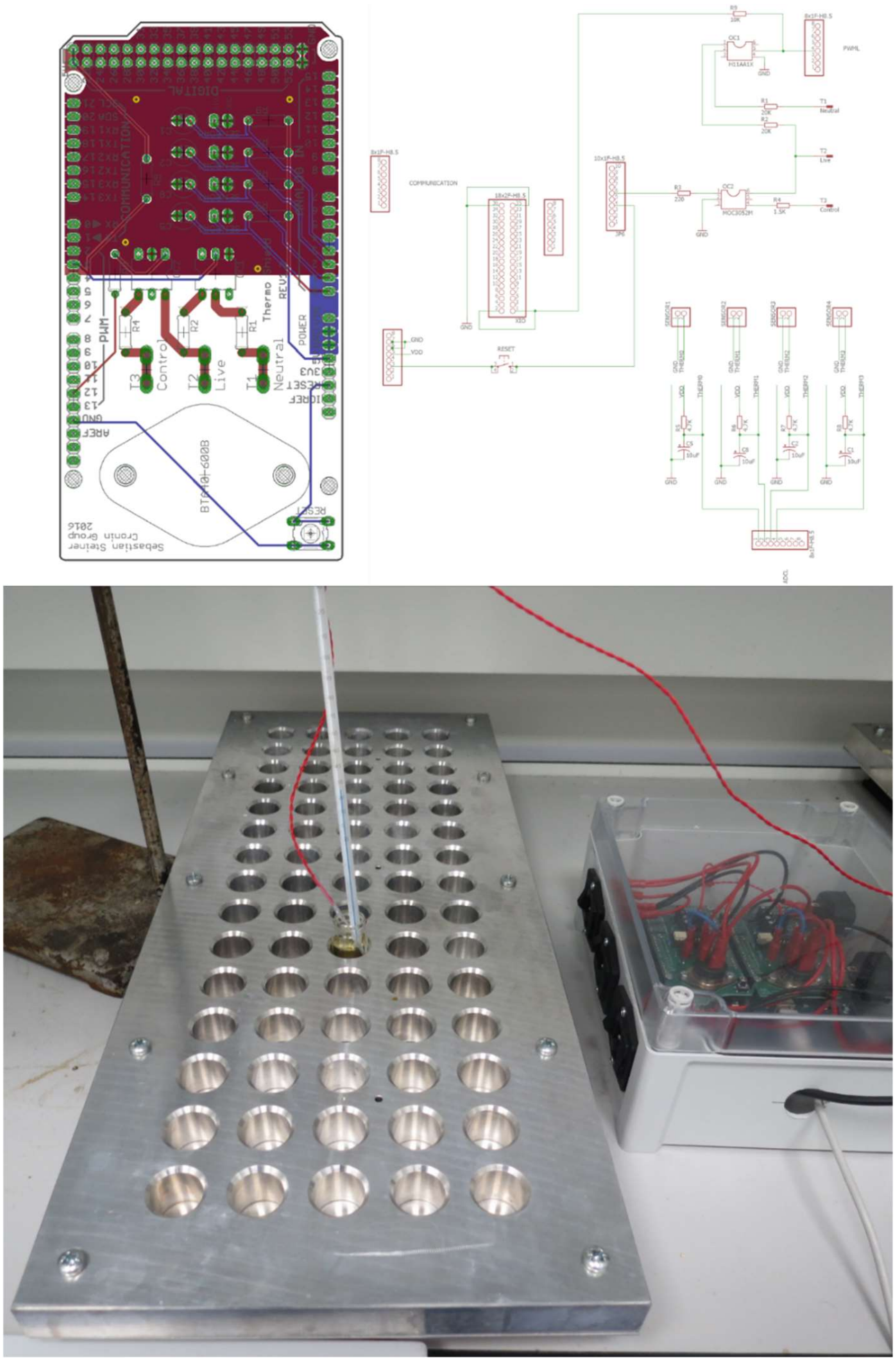

Figure S12: Temperature Control System for Wet-Dry Cycling Reactions. Thermistor was placed in a vial of mineral oil in the centre of the heating slab. Temperature was continuously monitored throughout the 15 hour heating cycles, through Termite and manually using a standard analogue thermometer inserted into the vial of mineral oil.
